# Ras interacting protein 1 facilitated proliferation and invasion of diffuse large B-cell lymphoma cells

**DOI:** 10.1080/15384047.2023.2193114

**Published:** 2023-03-26

**Authors:** Xiaojing Xing, Xuguang Wang, Meichen Liu, Qianxue Guo, Hongyue Wang

**Affiliations:** a Department of Hematology and Breast Cancer, Cancer Hospital of China Medical University (Liaoning Cancer Hospital & Institute), Shenyang, China; b Department of Pathology, Shenyang Medical College, Shenyang, China; c Department of Scientific Research and Academic, Cancer Hospital of China Medical University (Liaoning Cancer Hospital & Institute), Shenyang, China

**Keywords:** DLBCL, RASIP1, FOXO3, proliferation, invasion

## Abstract

A GTPase binding protein, Ras interacting protein 1 (RASIP1), has been reported with a tumor-promoting role in lung cancer cells, and its role in lymphoma remains unknown. The analysis of medical databank shows that RASIP1 is upregulated in diffuse large B-cell lymphoma (DLBCL) specimens. In this article, we demonstrated that RASIP1 is highly expressed in DLBCL cell lines, compared with primary B cells. The gain- and loss-of-function experiments were performed to investigate the effects of RASIP1 on DLBCL cells. CCK-8, flow cytometry, western blot, and transwell assays demonstrated that silence of RASIP1 inhibited proliferation, cell cycle transition, and invasion and induced significant apoptosis in DLBCL cells, and ectopic expression of RASIP1 played opposite roles. Xenograft results revealed that RASIP1 facilitated the growth of DLBCL cells *in vivo*. These findings suggest that RASIP1 may be required for malignancy of DLBCL cells. In addition, we also found that the expression of RASIP1 was negatively regulated by forkhead box O3 (FOXO3), which has been reported to suppress the proliferation of DLBCL cells. Our results indicate that FOXO3 is bound to the promoter sequence of RASIP1 and inhibits its transcription. The suppressive effects of FOXO3 on proliferation and invasion of DLBCL cells were neutralized by RASIP1. In conclusion, we demonstrate that FOXO3 negatively regulated RASIP1 facilitates growth and invasion of DLBCL cells, provides novel diagnostic markers and therapeutic targets for DLBCL in clinic.

## Introduction

Diffuse large B-cell lymphoma (DLBCL) is the most common lymphoma, accounting for 30% of non-Hodgkin’s lymphoma. Approximately 150,000 new cases are diagnosed annually worldwide^1^. DLBCL is characterized by heterogeneity, aggressiveness, and frequent relapse or resistance to chemotherapy^2^. Due to the heterogeneity of DLBCL, the immunological, pathological, molecular, and genetic characteristics of patients are quite diverse, which results in different outcomes^3–6^. The expression of several genes has been demonstrated to be associated with the survival of DLBCL cases, such as *LMO2*, *BCL6*, *CCND2*, and *BCL2*^7–10^. For instance, high BCL6 expression predicts better prognosis and positive expression of CCND2 is correlated with a shorter progression-free survival of DLBCL patients^8,9^. Additionally, the abnormal expression of several molecules provides references for classification of DLBCL, such as CD10, Bcl-6 and MUM1. The authors define the germinal center (GC) phenotype based on the combined expression of CD10 and Bcl-6 and define the non-GC phenotype as MUM1 positive and either or both negative for Bcl-6 and CD10^11^. Therefore, it is beneficial for diagnosis, classification, and treatment to determine the expression of some molecular markers and explore their underlying mechanism.

**Figure 1.**
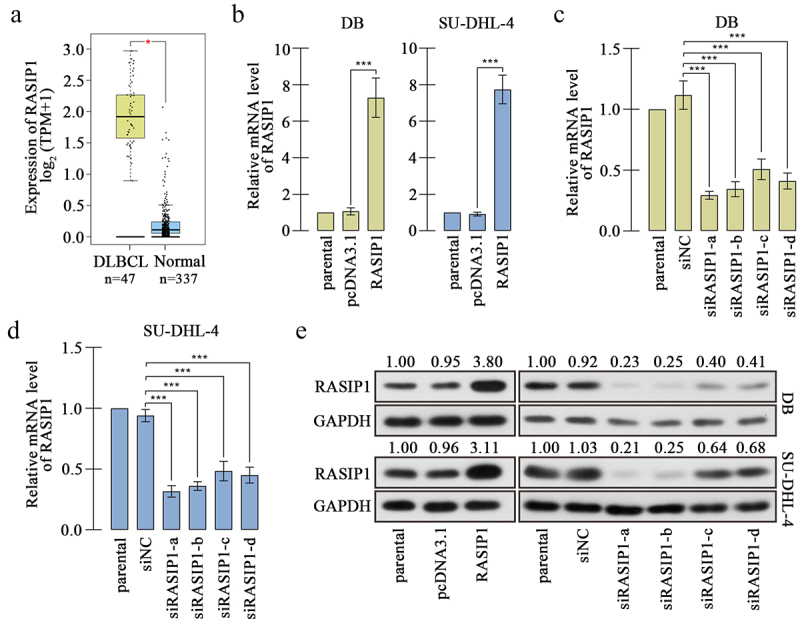
RASIP1 was increased in DLBCL specimens.

Ras interacting protein 1 (RASIP1) is a GTPase binding protein and firstly identified in 2004^12^. RASIP1 contains a Ras-associating domain, through which RASIP1 binds to GTP-loaded form of Ras and mediates the Ras-GTP downstream signaling cascades.^13^ Subsequent reports demonstrate that RASIP1 is an endothelial-specific protein that plays a key role in vascular development and angiogenesis via regulating cell–cell junctions^14–16^. Recent studies have found that RASIP1 controls the motility of not only endothelial cells but also some tumor cells, such as lung cancer^17,18^, suggesting that RASIP1 may be involved in the malignancies of cancer cells. However, the effects of RASIP1 on DLBCL cells have not been elucidated. DLBCL is a type of aggressive tumor, accompanied by frequent extranodal dissemination and filtration of lymphoma cells^19^. Analysis from the bioinformatics website Gene Expression Profiling Interactive Analysis (GEPIA) (http://gepia2.cancer-pku.cn/#index) shows that RASIP1 is highly expressed in clinical DLBCL specimens, compared with normal blood samples (shown in Figure 1a), suggesting that RASIP1 may serve as a tumorigenic molecule during DLBCL development. In addition to metastasize, uncontrolled proliferation is another hand of malignancy tumor cells. However, the effects of RASIP1 on cell growth and proliferation have not been reported yet.

(a) The medical databank GEPIA showed that RASIP1 was upregulated in DLBCL specimens. (b-d) Real-time PCR was used to confirm the effectiveness of overexpression plasmid (b) and interference RNA in RASIP1 at DB (c) and SU-DHL-4 cells (d) at mRNA level. (e) The effectiveness of overexpression or silencing of RASIP1 in DB and SU-DHL-4 cells was verified at protein level by western blot. (***p < 0.001)

Moreover, we also found that the expression of RASIP1 is regulated by forkhead box O3 (FOXO3), which has been reported with a cancer suppressive role in multiple tumors, including DLBCL^20^. FOXO3 generally acts as a transcription factor activating or suppressing the transcription of downstream genes^21,22^. In this study, we demonstrated that the expression of RASIP1 was negatively regulated by FOXO3 *in vitro*. We hypothesized that FOXO3 may regulate the malignancy behavior of DLBCL cells by controlling the transcription of RASIP1. In the present study, we tried to illustrate that 1) whether and how RASIP1 affects the proliferation and invasion of DLBCL cells; 2) whether FOXO3 regulates the malignancy behavior of DLBCL by controlling RASIP1 expression.

## Material and methods

### Cell culture and transfection

Human DLBCL cell line DB was purchased from Procell Life Science&Technology Co., Ltd. (Wuhan, Hubei, China), and SU-DHL-4 from Cellcook Life Science&Technology Co., Ltd. (Guangzhou, Guangdong, China). The DB and SU-DHL-4 cells were cultured with RPMI-1640 containing 10% fetal bovine serum (FBS) in a humid thermostatic incubator with 37°C and 5% CO_2_. The DB cell line was established by Walter J. Urba and Dan L. Longo from ascites fluid from a male patient with a diffuse large cell lymphoma in 1990^23^. SU-DHL-4 was a lymphoblast-like cell line that was isolated from the peritoneal effusion of a male patient by A L. Epstein in 1976^24^. The DB and SU-DHL-4 cells were cultured in suspension, and the details of these two cell lines can be seen in ATCC (https://www.atcc.org/).

Human primary B cells from peripheral blood were purchased from iCell Bioscience Inc. (Shanghai, China) and cultured with B lymphocyte-specific medium (iCell Bioscience Inc.) supplemented with 10% FBS. The human embryonic kidney cell-line 293T was purchased from Zhong Qiao Xin Zhou Biotechnology Co., Ltd. (Shanghai, China) and cultured with DMEM containing 10% FBS. 293T cells served as tool cells for dual-luciferase reporter assay.

To control the level of RASIP1 or FOXO3, DB and SU-DHL-4 cells were transfected with the overexpression vector or interference RNA fragment of RASIP1 or FOXO3 in the presence of Lipo8000 reagent (Beyotime, Shanghai, China). PcDNA3.1 empty vector or negative control interference RNA (siNC) served as the control.

To obtain the RASIP1-stably silenced cell line, the RASIP1 knockdown plasmid was transfected into DB and SU-DHL-4 cells, which underwent G418 (200 μg/ml) treatment for about 6 weeks. The live cells were considered with stably low expression of RASIP1 and used for xenograft experiments.

### Real-time PCR

The total RNA was extracted with TRIpure lysis buffer (BioTeke Corporation Co., Ltd., Beijing, China), and the concentration was determined by a NANO2000 ultraviolet spectrophotometer (Thermo Fisher Scientific Co., Ltd., Waltham, MA, USA). Subsequently, the reverse transcription (RT) was performed using BeyoRT II M-MLV reverse transcriptase (Beyotime) with oligo(dT) and random primer as RT primers. The obtained cDNA was used for real-time PCR to determine the mRNA level of FOXO3 and RASIP1, in the presence of 2× Taq PCR MasterMix and SYBR Green (Solarbio, Beijing, China), and GAPDH served as the internal control. The PCR procedure was set as follows: 94°C for 5 min 10 s, 60°C for 20 s, 72°C for 30 s, followed with 40 cycles of 72°C for 2 min 30 s, 40°C for 1 min 30 s, melting 60–94°C, every 1°C for 1 s, and finally incubated at 25°C for several minutes. The data were calculated using 2^−ΔΔCT^ method. The primers were synthesized by General Biology Co., Ltd. (Chuzhou, Anhui, China), and the sequence information is shown in Table 1.

**Table 1.** The sequence information of PCR primers.

Name	Sequence	Tm	Length of Amplification	Gene ID
FOXO3 forward	5’-TGACGACAGTCCCTCCC-3’	52.9°C	112 bp	NM_001455
FOXO3 reverse	5’-GCTGGCGTTAGAATTGGT-3’	53.2°C		
RASIP1 forward	5’-TCAACTCGCTGATGGAACG-3’	57.4°C	136 bp	NM_017805
RASIP1 reverse	5’-AAGAACTCAGTGGCAATGTCG-3’	57.7°C		
ChIP RASIP1 forward	5’-ACCACGGAGTCGTCCATAA-3’	55.6°C	151 bp	
ChIP RASIP1 reverse	5’-CAAGCCAAGCCTGTCTTCT-3’	55.1°C		

### Western blot

The total protein was extracted with a RIPA lysis buffer, and its concentration was determined with a BCA kit (Solarbio). The polyacrylamide gel was prepared in advance, and its concentration was determined by the size of the protein to be detected. The protein sample was loaded into a polyacrylamide gel, followed by electrophoresis for 2–3 h. Afterward, the protein was transferred onto a polyvinylidene fluoride membrane (Millipore, Billerica, MA, USA), and skim milk was used to block the heterogenetic antigens. Subsequently, the membrane loaded with protein was incubated with primary antibody at 4°C in the dark overnight. After rinsing with TBST buffer, the membrane was incubated with a secondary antibody labeled with HRP, and reacted with ECL reagent (Solarbio) for several minutes, followed by signal exposure in the dark. The antibody information was shown in the following: RASIP1 (1:1000; Affinity, Changzhou, Jiangsu, China), cyclin E1 (1:1000; Affinity), cyclin B1 (1:1000; Affinity), cyclin D1 (1:1000; Affinity), p27 (1:1000; Affinity), matrix metalloproteinase 2 (MMP2) (1:2000; Novus Biologicals, Littleton, CO, USA), MMP9 (1:1000; Affinity), cleaved caspase-3 (1:1000; Affinity), Bax (1:1000; Affinity), Bad (1:1000; Affinity), Bcl-xl (1:1000; Affinity), FOXO3 (1:500; Affinity), GAPDH (1:10000; Affinity).

### CCK-8 assay

The DB or SU-DHL-4 cells were seeded into 96-well plates and cultured for certain periods of time. The cells were then treated with CCK-8 reagent (Biosharp, Hefei, China) with 10 μl per well for 2 h. Thereafter, the optical density (OD) at 450 nm of the medium was measured with a microplate reader.

### Flow cytometry

Flow cytometry was used to measure the cell cycle and apoptosis of DB and SU-DHL-4 cells.

For the cell cycle determination, the cells were collected and fixed with 70% ethanol at 4°C for 2 h. The cells were then stained with propidium iodide (PI) (Biosharp) at 37°C for 30 min in the dark. Subsequently, the cells were used for flow cytometry to analyze the distribution of cells in each phase.

For apoptosis detection, the cells were collected and stained with Annexin V-FITC at room temperature for 10 min, followed by the incubation with PI for 5 min. Finally, the apoptosis was analyzed with a flow cytometer (ACEA Biosciences Inc., San Diego, CA, USA). Annexin V+PI- cells were considered as early apoptotic cells, and Annexin V+PI+ cells as late apoptotic cells.

### Transwell assay

To measure the invasive ability of DB and SU-DHL-4 cells, the transwell assay was performed. The transwell chambers (Labselect, Hefei, Anhui, China) were pre-coated with matrigel (Corning, NY, USA) for 2 h. After counting, the cells were seeded into the upper chambers with a serum-free medium, and the lower chambers were added with medium containing 10% FBS. The cells were cultured in the transwell chambers for 24 h, and then the number of cells on the reverse surface of the chambers was counted.

### Dual-luciferase reporter assay

To confirm the binding between FOXO3 and promoter sequence of RASIP1, dual-luciferase reporter assay was carried out. Four sequences with different length (−1755/+15, −1237/+15, −802/+15 and −486/+15) were cloned into pGL3 vector, which was co-transfected into 293T cells with FOXO3 and pRL-TK vector. After 48 h, the cells were treated with a Dual Luciferase Reporter Gene Assay Kit according to the manufacturer’s protocol, and the Firefly and Renilla values were measured with a microplate reader (BioTek, Winooski, VT, USA).

### Chromatin immunoprecipitation (ChIP)

ChIP was used to confirm the binding between FOXO3 and the sequence of RASIP1 promoters. DB or SU-DHL-4 cells underwent crosslinking with 1% formaldehyde for 10 min, terminated with the addition of glycine. Subsequently, the cells were treated with a ChIP Assay Kit (Beyotime) according to the manufacturer’s protocol. Briefly, the cells were lysed with ultrasonication, and the lysate was incubated with antibody-bound beads at 4°C for several hours. The DNA–protein complex was used for elution and de-crosslinking, and DNA was collected for PCR and agarose gel electrophoresis. The information of PCR primers is shown in Table 1. The sample before incubation with beads served as input control.

### Xenograft model

Healthy BALB/C nude mice (4–6 weeks) were maintained in a pathogen-free condition with free access to water and food. The mice were randomly divided into three groups: shNC, shRASIP1–1, and shRASIP1–2 (*n* = 6 per group). 1 × 10^7^ DB or SU-DHL-4 cells with stable silencing of RASIP1 or NC were mixed with matrigel and subcutaneously injected into mice. The size of the subcutaneous nodules was determined every 3 d. At 28 d post-inoculation, the mice were euthanized, and the tumors were isolated for subsequent examinations.

The animal feeding and experiments were performed according to the Guide for the Care and Use of Laboratory Animals (eightth edition), and the procedure was approved by the ethics committee of Shenyang Medical College (SYYXY2021080101).

### HE staining

The tumor tissues were fixed with 4% paraformaldehyde overnight and washed with flowing water for 4 h. Then, dehydration with grading concentrations of ethanol (70% for 2 h, 80% overnight, 90% for 2 h, 100% for 1 h twice), and hyalinization of xylene (for 30 min) were performed. Subsequently, the tissues were embedded into paraffin, and cut into 5-μm sections, which were deparaffinized with xylene and ethanol. The nuclei were stained with hematoxylin (Sinopharm, Shanghai, China), and cytoplasm was stained with eosin (Sangon Biotech (Shanghai) Co., Ltd., Shanghai, China). Finally, the sections were dehydrated again, mounted with gum, and photographed with a microscope (Olympus, Tokyo, Japan) at 200× magnification.

### Immunohistochemistry staining

The tumor tissues were made into paraffin sections as previously described. The sections reacted with antigen retrieval buffer in boiling for 10 min, followed by blocking with 3% H_2_O_2_ for 10 min and 1% BSA for 15 min. Subsequently, the sections were incubated with antibody against Ki-67 (1:50; Affinity) at 4°C overnight, and HRP-labeled secondary antibody (1:500; Thermo Fisher Scientific Co., Ltd.) at 37°C for 60 min. The sections then reacted with DAB reagents for several minutes and were counterstained with hematoxylin. Finally, the sections were dehydrated with ethanol and xylene, mounted with gum, and photographed with a microscope at 400× magnification.

### TUNEL assay

The tumor tissues were made into paraffin sections as in the previous description. After deparaffinization, the sections were permeated with 0.1% TritionX-100 (Beyotime) for 8 min, incubated with TUNEL reaction solution (Roche, Basel, Switzerland) in the dark for 60 min and counterstained with DAPI (Aladdin Biochemical Technology Co., Ltd. in Shanghai, China) in the dark for 5 min. Finally, the sections were mounted with anti-fading reagents, and photographed at 400× magnification.

### Statistical analysis

The data in this study were presented as mean ± SD, and analyzed with GraphPad Prism 7.0. Data from two independent groups were analyzed with student *t* test. The data from multiple groups were analyzed with one-way ANOVA followed by Bonferroni multiple comparisons. A *p* value less than 0.05 was considered to be statistically significant.

## Results

### RASIP1 promoted proliferation and cell cycle transition of DLBCL cells

Analysis from medical databank GEPIA exhibited that RASIP1 was upregulated in DLBCL specimens, compared with the normal blood specimens (the dataset sources were the Cancer Genome Atlas Program (TCGA) and Genotype Tissue Expression (GTEx) projects) (Figure 1a). We also demonstrated that RASIP1 mRNA and protein expression were increased in DLBCL cell lines, DB, and SU-DHL-4, compared with those in human primary B cells (Figure S1A and S1B). In order to study the function of RASIP1, its overexpression plasmid and four short interference RNAs (siRNAs) were synthesized and delivered into DB and SU-DHL-4 cells. Real-time PCR and western blot confirmed that RASIP1 was increased by more than 7 folds after transfection of the ectopic expression plasmid and decreased by more than 50% after transfection of siRNA in DB and SU-DHL-4 cells, compared with empty vector or siNC control (Figure 1b-e). Two siRNAs with better silencing effectiveness were selected for the subsequent experiments.

**Figure 2.**
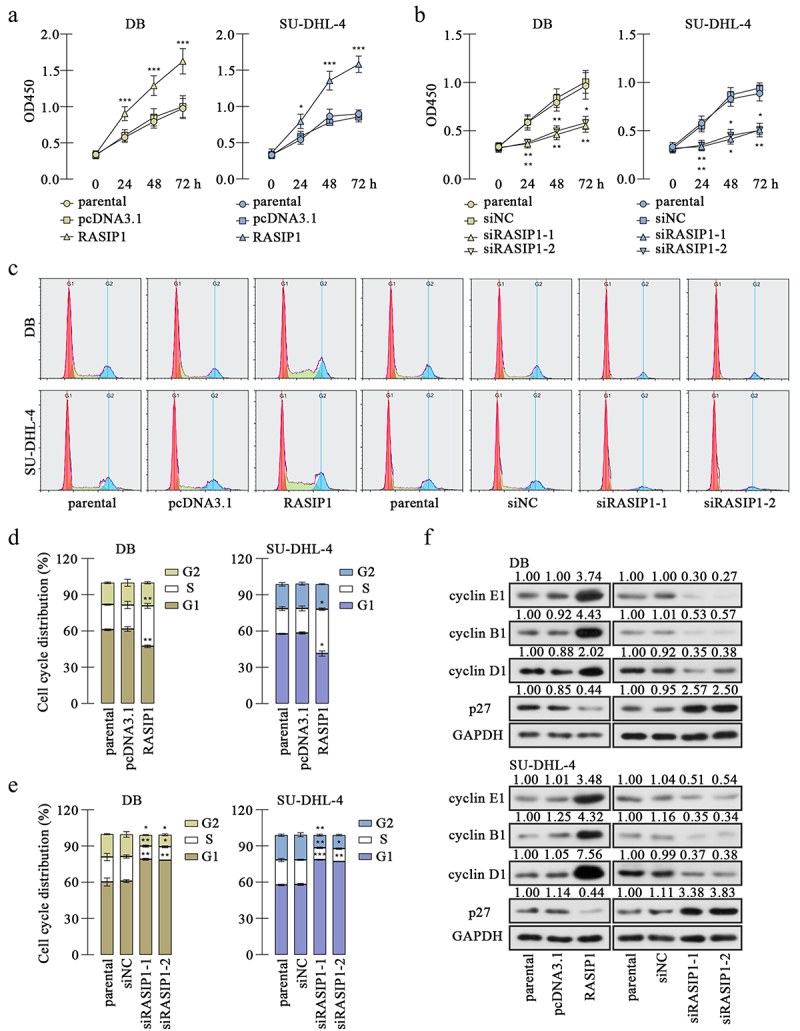
RASIP1 promoted proliferation and cell cycle transition of DLBCL cells.

CCK-8 assay revealed that RASIP1 overexpression promoted proliferation, which was suppressed after RASIP1 knockdown in DB and SU-DHL-4 cells Figure 2(a,b). Flow cytometry results showed that RASIP1 accelerates the G1/S transition and its silence delayed G1/S and G2/M progress in DB and SU-DHL-4 cells (Figure 2c-e). Thereafter, western blot results displayed that the expression of cell cycle – related protein, cyclin E, cyclin B1, and cyclin D1 was increased, and the inhibitor of cyclin-dependent kinase p27 was reduced after RASIP1 overexpression, compared with the empty vector control (Figure 2f). Meanwhile, after RASIP1 knockdown, the expression of cyclin E, cyclin B1, and cyclin D1 was decreased and that of p27 was increased (Figure 2F). The results in this section suggest that RASIP1 enhances proliferation and cell cycle G1/S transitions of DLBCL cells.

(a) and (b) CCK-8 assay was applied to determine the viability of DB and SU-DHL-4 cells with enhanced expression or knockdown of RASIP1. (c-e) The distribution of DB and SU-DHL-4 cells in each phase was determined by flow cytometry. (f) The expression levels of several cell cycle-related molecules, cyclin E, cyclin B1, cyclin D1, and p27 were examined by immunoblotting. (*p < 0.05, **p < 0.01, ***p < 0.001 vs pcDNA3.1 or siNC group)

### The RASIP1 silencing facilitated apoptosis of DLBCL cells

**Figure 3.**
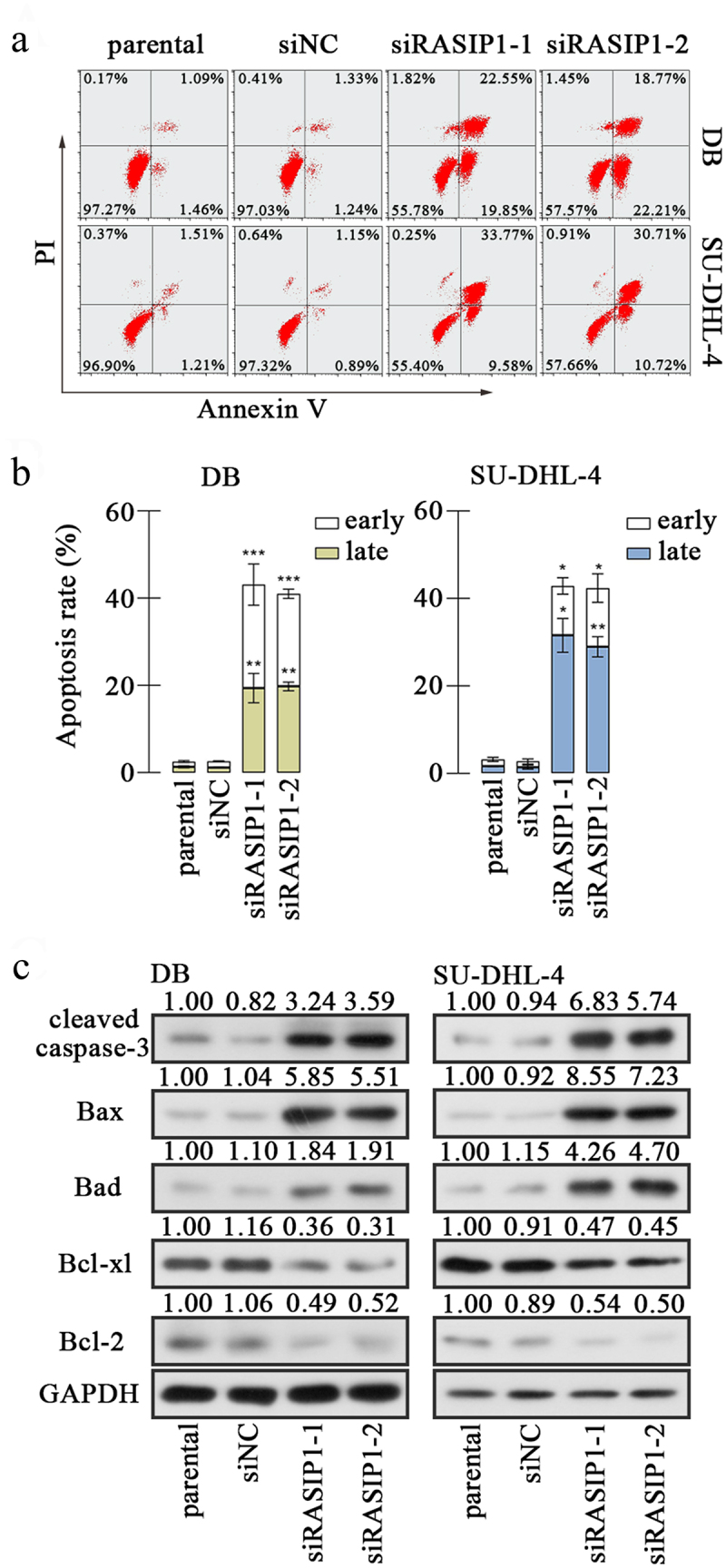
The RASIP1 silencing facilitated apoptosis of DLBCL cells.^1^1.There is an error in Figure 3, and the revised figure 3 is re-uploaded.

Subsequently, the apoptosis of DB and SU-DHL-4 cells was assessed with flow cytometry after RASIP1 silencing. Annexin V+ was considered a marker of early apoptosis, and PI+ was considered as a marker of late apoptosis and necrosis. Therefore, Annexin V+PI- cells were counted as early apoptotic cells, and Annexin V+PI+ cells as late apoptotic cells. The results revealed that the silence of RASIP1-induced apoptosis of DB and SU-DHL-4 cells, including both early and late apoptosis Figure 3a,b. Thereafter, the levels of several apoptosis-related molecules were examined. Data from Western blot showed that the levels of pro-apoptotic proteins, cleaved caspase-3, Bax, and Bad, were elevated, and that of anti-apoptotic proteins, Bcl-xl and Bcl-2, were reduced in RASIP1-silenced DB and SU-DHL-4 cells (Figure 3c). These results demonstrate that apoptosis of DLBCL cells is induced when RASIP1 expression is inhibited.

(a) and (b) The apoptosis of DB and SU-DHL-4 cells with RASIP1 knockdown was detected by flow cytometry via Annexin V and PI staining. (c) The levels of apoptosis-related molecules, cleaved caspase-3, Bax, Bad, Bcl-xl, and Bcl-2, were determined by western blot. (*p < 0.05, **p < 0.01, ***p < 0.001 vs siNC group)

### RASIP1 enhanced invasion of DLBCL cells

**Figure 4.**
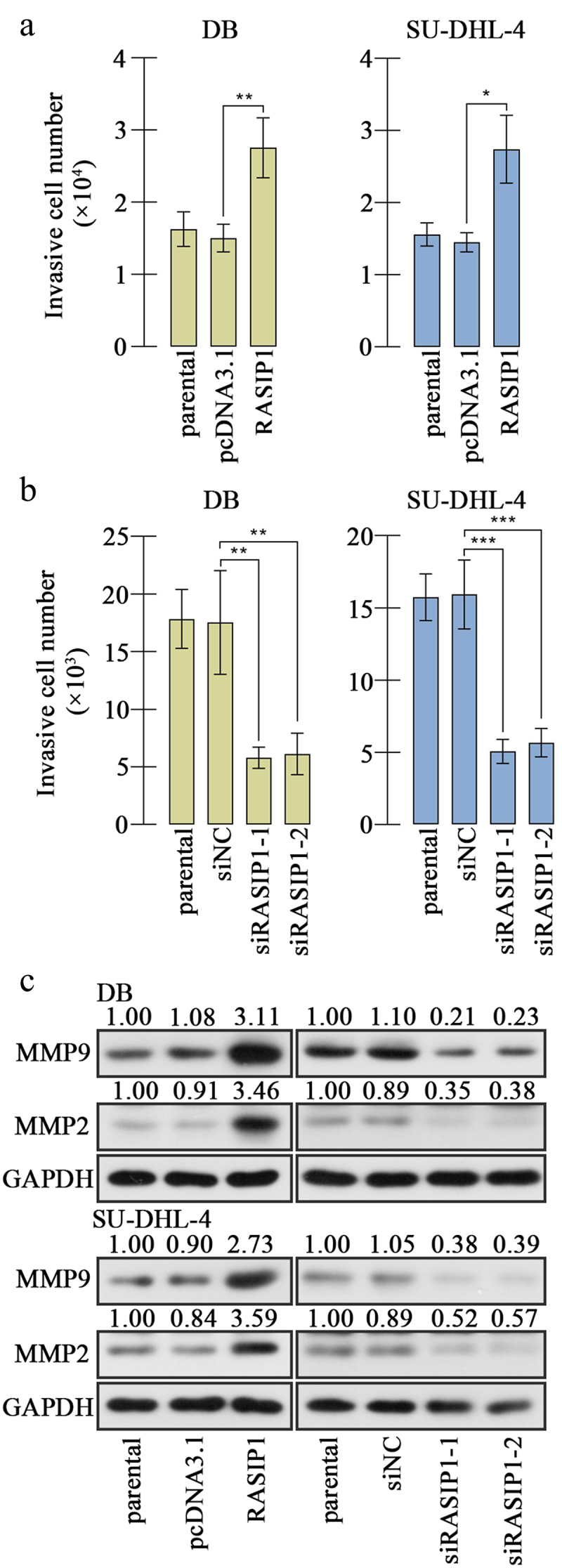
RASIP1 enhanced the invasion of DLBCL cells.

Considering filtration as a vital aspect of malignant tumors, we evaluated the invasion of DLBCL cells. The transwell assay results revealed that the invasive ability of DB and SU-DHL-4 cells was strengthened by RASIP1 overexpression and attenuated by RASIP1 knockdown Figure 4(a,b). The levels of mature MMP2 and MMP9 were raised after ectopic expression of RASIP1 and receded after knockdown of RASIP1 in DB and SU-DHL-4 cells (Figure 4c). The results in this section suggest that RASIP1 aggravated invasion of DLBCL cells.

(a-b) Transwell assay with matrigel was carried out to evaluate the invasive ability of DB and SU-DHL-4 cells. (c) The levels of mature MMP2 and MMP9 were detected. (*p < 0.05, **p < 0.01, ***p < 0.001 vs pcDNA3.1 or siNC group)

### RASIP1 was transcriptional repressed by FOXO3

**Figure 5.**
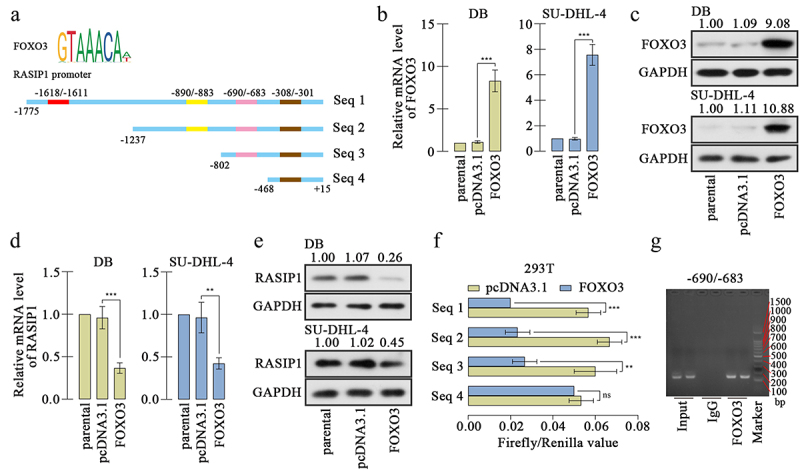
RASIP1 was transcriptional repressed by FOXO3.

**Figure 6.**
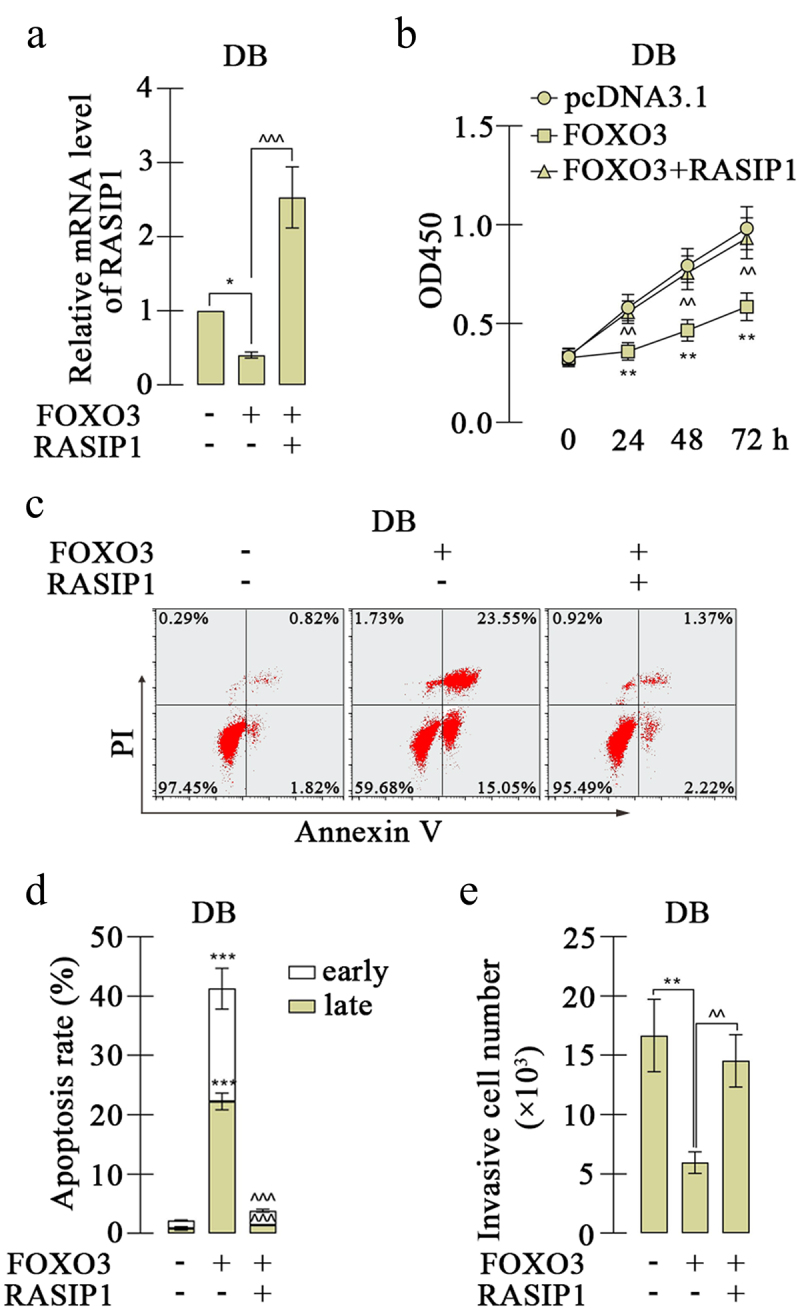
The effects of FOXO3 on proliferation and apoptosis of DLBCL cells were reversed by RASIP1.

The analysis from bioinformatics website JASPAR (https://jaspar.genereg.net/) exhibited that the promoter sequence of RASIP1 was potentially bound by FOXO3, and there were four possible sites (Figure 5a). To investigate the effects of FOXO3, a FOXO3 overexpression plasmid was constructed and transfected into DB and SU-DHL-4 cells. The high expression of FOXO3 was confirmed by real-time PCR and western blot (Figure 5(b,c). FOXO3 expression caused downregulation of RASIP1 expression in both mRNA and protein levels in DB and SU-DHL-4 cells Figure 5(d,e). Meanwhile, the silencing of FOXO3 increased the expression of RASIP1 (Figure S2A-D). To explore the binding details between FOXO3 and promoter sequence of RASIP1, four promoter sequence fragments containing four or three or two or one binding sites were cloned into luciferase reporter vector (as shown in Figure 5a). The luciferase reporter's assay revealed that when the −690/-683 site was removed, the inhibition effects of FOXO3 on luciferase activity of vector that contains RASIP1 were significantly abrogated (Figure 5f). Therefore, a ChIP assay was performed to confirm the binding between FOXO3 and −690/-683 site in the RASIP1 promoter sequence. The results of agarose gel electrophoresis demonstrated that FOXO3 certainly bound to −690/-683 site in RASIP1 promoter sequence (Figure 5g). To verify whether the tumor-promoting roles of RASIP1 were regulated by FOXO3, both FOXO3 and RASIP1 were overexpressed in DB cells. The RASIP1 overexpression plasmid only contained RASIP1 coding sequence, but not its promoter sequence. So the exogenous expression of RASIP1 was not regulated by FOXO3. Real-time PCR results displayed that FOXO3-induced reduction of RASIP1 was significantly reversed by its exogenous overexpression (Figure 6a). CCK-8 assay showed that FOXO3-inhibited proliferation of DB cells was recovered by RASIP1 (Figure 6b). Moreover, FOXO3 caused significant early and late apoptosis in DB cells, which were abolished by RASIP1 Figure 6(c,d). Similarly, FOXO3-suppressed invasive ability of DB cells was partially rescued by RASIP1 (Figure 6e). The results in this section suggest that FOXO3 is bound to the promoter sequence of RASIP1, and negatively regulates its expression in DLBCL cells. These results supported that FOXO3 may play its anti-lymphoma role by downregulating RASIP1.

(a) The transcription factor FOXO3 potentially bound to four sites in RASIP1 promoter sequences. (b) and (c) The effectiveness of FOXO3 overexpression plasmid was confirmed in DB and SU-DHL-4 cells. (d) and (e) The expression of RASIP1 in DB and SU-DHL-4 cells after FOXO3 overexpression was determined at transcription and translation levels. (f) Dual-luciferase reporter assay was performed to confirm the binding between FOXO3 and the potential sites in RASIP1 promoter sequence. (g) ChIP assay was used to verify the binding between FOXO3 and the -690/-683 site in RASIP1 promoter sequence. (**p < 0.01, ***p < 0.001, ns, no significance vs pcDNA3.1 group)

(a) The expression of RASIP1 in DB cells with FOXO3 and/or RASIP1 overexpression was determined by real-time PCR. (b) The viability of DB cells was measured by CCK-8 assay. (c) and (d) Apoptosis of DB cells with FOXO3 and/or RASIP1. (e) The invasion of DB cells was measured. (*p < 0.05, **p < 0.01, ***p < 0.001 vs pcDNA3.1 group; ^^p < 0.01, ^^^p < 0.001 vs FOXO3 + pcDNA3.1 group)

### RASIP1 enhanced the growth of DLBCL cells in vivo

**Figure 7.**
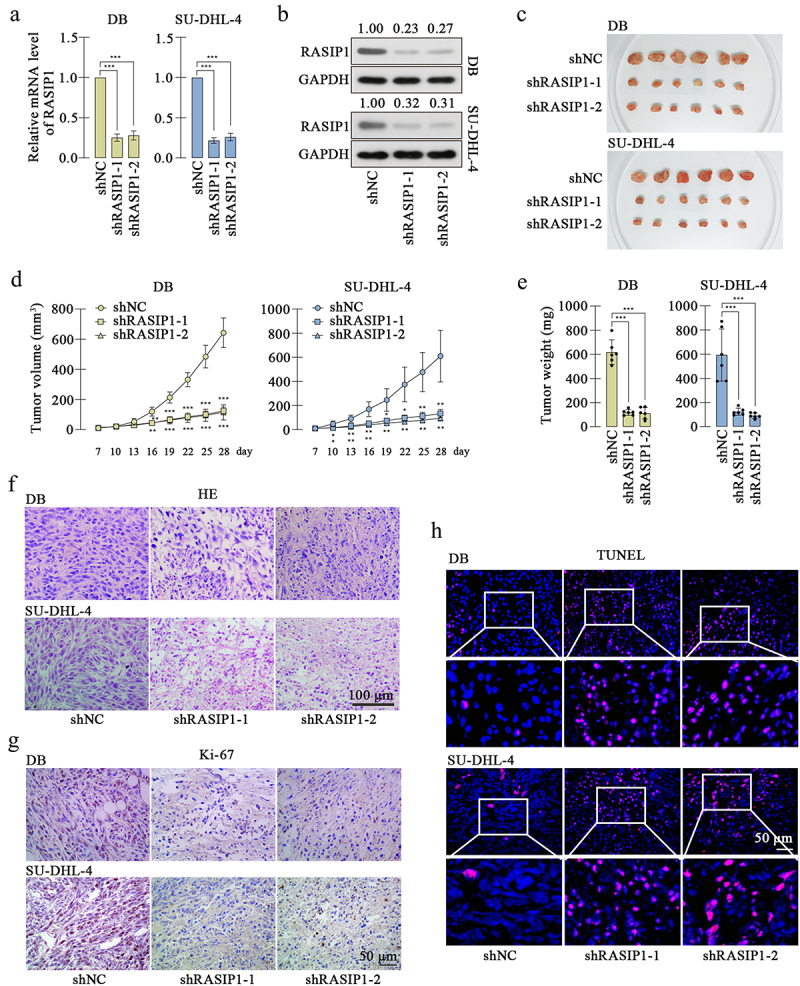
RASIP1 knockdown inhibited the growth of DLBCL cells *in vivo*.

To further confirm the effects of RASIP1 on DLBCL cells *in vivo*, xenograft model was induced in nude mice. The DB or SU-DHL-4 cells with stable knockdown of RASIP1 were constructed, and the knockdown effectiveness was verified at transcription and translation levels Figure 7(a,b). After subcutaneous inoculation for 28 d, the tumors were isolated. Figure 7(c,d) showed that the growth of RASIP1-silenced DB or SU-DHL-4 cells was significantly suppressed. HE staining displayed that the siRASIP1 led to some pathological alterations in tumors, including nuclear shrinking and interstice among cells (Figure 7f). Immunohistochemistry staining exhibited that the expression of the proliferation marker Ki-67 was significantly reduced after RASIP1 knockdown (Figure 7g). Similarly, aggravated apoptosis was found in RASIP1-silenced tumors by TUNEL staining (Figure 7h). The results in this section confirmed that the suppressed RASIP1 expression induced the growth limitation of DLBCL cells *in vivo*.

The RASIP1-stably silenced DB and SU-DHL-4 cell lines were constructed, and real-time PCR (a) and western blot (b) were performed to confirm the knockdown effects. (c) The subcutaneous tumors formed by RASIP1-silenced DB or SU-DHL-4 cells. (d) The growth curve of tumors. (e) The tumor weights. (f) HE staining was used to display the pathological alterations of tumors after RASIP1 knockdown. (g) Immunohistochemical staining of Ki-67. (h) TUNEL assay was performed to measure cell apoptosis in tumors. (*p < 0.05, **p < 0.01, ***p < 0.001 vs shNC group)

## Discussion

The data in the present study demonstrated that RASIP1 promoted the proliferation and cell cycle transition of DLBCL cells. Proliferation depends on the cell cycle transition progress, and cancer cells are characterized by uncontrolled proliferation and aberrant cell cycle activity^25^. The overexpression of RASIP1 caused a significant increase of G1/S checkpoint markers, cyclin E1 and D1^26^, and a decrease of p27, an inhibitor of cyclin E-cyclin-dependent kinase (CDK) 2 and cyclin D-CDK4/6^26^, accompanied with the reduced G1 phase distribution and elevated S phase distribution of DLBCL cells. RASIP1 silencing induced opposite results, suggesting that RASIP1 accelerated the G1/S transition of DLBCL cells. However, there seems to be a contradiction in the G2/M transition. RASIP1 knockdown decreased the G2 phase distribution and the expression of a G2/M checkpoint marker, cyclin B1^26^, and RASIP1 overexpression only promoted the expression of cyclin B1, but did not affect the proportion of cells in G2 phase. It is well known that flow cytometry analyzed cell cycle phases by determining DNA content. The DNA content is doubled in prophase, metaphase, and anaphase, so some cells in M phase may be mistaken for G2-phase cells. Combining CCK-8, flow cytometry, and western blot results, we conclude that the proliferation and whole-cell cycle transition of DLBCL cells were accelerated by RASIP1.

Proliferation and apoptosis are intimately coupled. In cancer tissues, the balance between proliferation and programmed cell death is broken, and apoptosis is largely suppressed. In mammalian cells, the extrinsic death signals deliver through death receptors to active caspase-8, which is an initiator of caspase.^27,28^. The intrinsic apoptosis is initiated by mitochondrial pathway, including increased mitochondrial permeability and release of cytochrome C into cytoplasm, which promoted the activation of caspase-9^29^. Both cleaved caspase-8 and cleaved caspase-9 activate caspase-3, which is a well-known apoptosis executioner to mediate downstream apoptotic signaling^30^. Moreover, the intrinsic apoptosis is regulated by Bcl-2 family proteins, including pro-apoptotic members such as Bax, Bad, Bid, Bik, Bim, and Bcl-xs, and anti-apoptotic members such as Bcl-2, Bcl-xl, and Bcl-w^31^. In our study, RASIP1 knockdown induced the increased expression of activated caspase-3, Bax, and Bad and decreased expression of Bcl-xl and Bcl-2, suggesting that RASIP1 silencing resulted in intrinsic apoptosis in DLBCL cells. Notably, a t(14:18) translocation leads to a rearrangement in the *BCL2* gene, resulting in promoted expression of Bcl-2 by both transcriptional activation and abnormal posttranscriptional regulation of Bcl-2 mRNA in SU-DHL-4 cells^32^. As an anti-apoptotic protein, Bcl-2 targets mitochondria to prevent apoptosis initiation through cytochrome C release^33^, and also directly binds inositol 1,4,5-trisphosphate receptors and suppresses their activity to prevent the pro-apoptotic Ca^2+^ flux from endoplasmic reticulum into mitochondria^34^. The rearrangement of *the BCL2* gene endows cancer cells with anti-apoptotic ability, and Bcl-2 is considered a therapeutic target for tumor treatment. Our data demonstrate that RASIP1 significantly inhibited the protein level of Bcl-2 in both DB and SU-DHL-4 cells, suggesting that RASIP1 may participate in regulating Bcl-2 expression. However, the underlying details need to be illuminated by further investigation.

On the other hand, RASIP1 mainly functions by regulating the Ras downstream signaling as a Ras interacting protein. There are three isoforms of Ras with high sequence homology, including K-Ras, H-Ras, and N-Ras, among which K-Ras is the most commonly mutated isoform in cancer^35,36^. RASIP1 was first identified with a yeast two-hybrid screen using H-Ras protein as the bait. However, the sequence and structure information of RASIP1 indicated that it could interact with all Ras isoforms^12,13^. Ras proteins are considered as major drivers of human cancers, and Ras and its effectors are often regarded as cancer therapeutic targets. For instance, Apatinib, an oral chemotherapy agent, dramatically inhibits the growth of DLBCL cells by suppressing the Ras/Raf/MEK/ERK signaling^37^. A Ras/Raf pathway inhibitor, L744,832, induces cytotoxicity and cell death in DLBCL and Burkitt’s lymphoma cells.^38^ It is possible that RASIP1 plays its lymphoma-promoting role by interacting with Ras. Meanwhile, RASIP1 has been demonstrated to bind to Rap1^15^, and regulate Cdc42^39^, suggesting that RASIP1 may function by regulating other molecules. The interaction of RAS and Bcl-2 was demonstrated in cancer cells in the last century^40,41^. Therefore, it is reasonable to assume that RASIP1 may command Bcl-2 expression by interacting with RAS, and further regulate apoptosis of DB and SU-DHL-4 cells. This speculation needs to be confirmed by more experiments.

**Figure 8.**
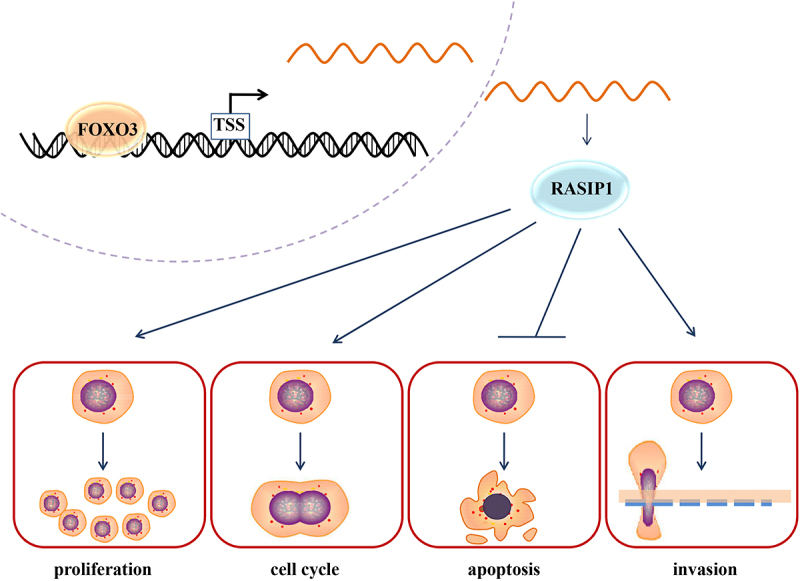
FOXO3-regulated RASIP1 promoted malignant behaviors of DLBCL cells.

In addition, our results demonstrated that the transcription of RASIP1 was negatively regulated by FOXO3, which has been reported as an anti-tumor factor in multiple cancer cells. A recent article reported that FOXO3 inhibited the proliferation of DLBCL cells, and its expression was negatively related with the 5-y prognosis and survival of DLBCL patients. In our results, FOXO3 was demonstrated to suppress the transcription of RASIP1 by binding to its promoter −690/-683 sequence, and the suppressive effects of FOXO3 on DLBCL cell behaviors were reversed by RASIP1. These data suggested that the tumor-promoting roles of RASIP1 were controlled by FOXO3 (Figure 8). The medical databank shows that FOXO3 is downregulated in DLBCL specimens (*p* > .05, data is not shown). Properly, the expression of RASIP1 and FOXO3 should be determined in DLBCL specimens and normal lymphoid tissue. However, the data of DLBCL-matched control from GTEx databank is obtained from normal blood samples. Due to the difficulty in acquiring healthy lymphoid tissues, we have not collected enough samples to perform the detection. The results for primary B cells and DLBCL cell lines suggest the high expression of RASIP1 in DLBCL cells. In our next plan, we will continue to collect the DLBCL and normal lymphoid specimens to determine the expression of RASIP1 and FOXO3, which would be reported in the subsequent papers.

FOXO3 is bound to the promoter sequence of RASIP and suppressed its transcription. RASIP1 enhanced proliferation, cell cycle transition, and invasion and inhibited apoptosis of DLBCL cells.

In conclusion, we found that RASIP1 was upregulated in DLBCL tissues and cell lines. The enhanced expression of RASIP1 promoted proliferation, cell cycle, invasion, and tumorigenesis, and suppressed apoptosis of DLBCL cells. The transcription of RASIP1 was inhibited by FOXO3, a lymphoma-suppressing transcription factor. The present study suggests RASIP1 as a potential therapeutic target of lymphoma.

## Supplementary Material

Supplemental Material

## Data Availability

The data sets used and/or analyzed during the current study are available from the corresponding authors on reasonable request.
