# Supplementary material for: Ras interacting protein 1 facilitated proliferation and invasion of diffuse large B-cell lymphoma cells
Source: Cancer Biol Ther. 2023 Mar 26;24(1):2193114. doi: 10.1080/15384047.2023.2193114 (PMC10054171; doi:10.1080/15384047.2023.2193114)
Supplement: Supplemental Material [file KCBT_A_2193114_SM5303.zip › supplementary.docx]

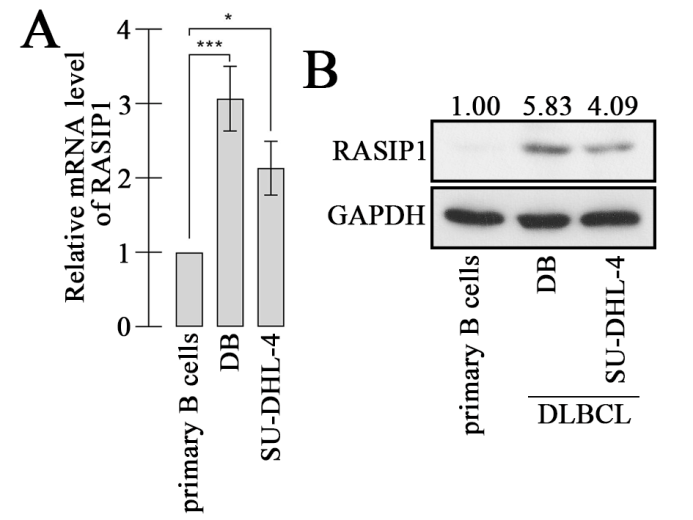


Figure S1. RASIP1 was highly expressed in DLBCL cell lines.

The mRNA (A) and protein (B) levels of RASIP1 in DLBCL cell lines, DB and SU-DHL-4, and human primary B cells were determined by real-time PCR and western blot.


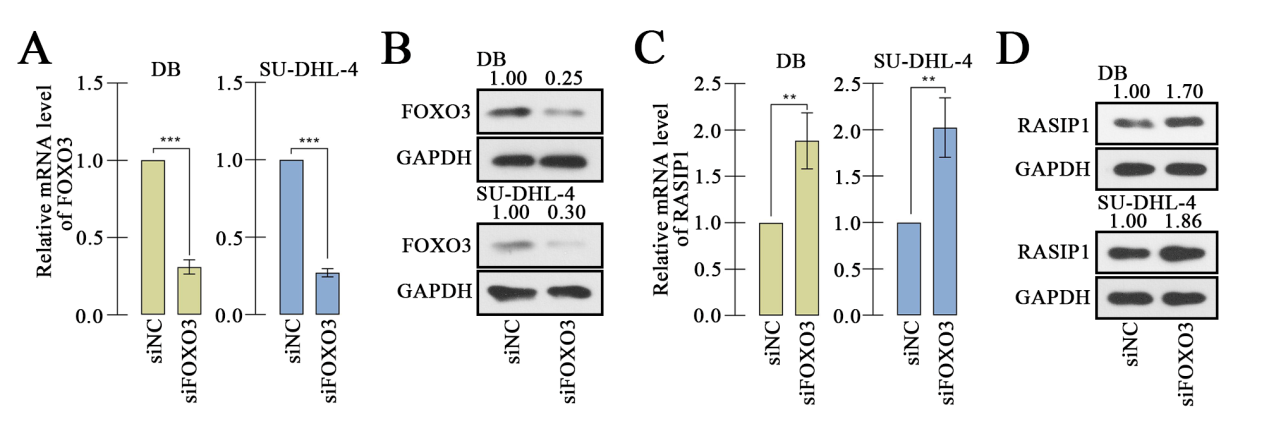


Figure S2. The silencing of FOXO3 increased the expression of RASIP1.

(A) and (B) Real-time PCR and western blot were used to confirm the knockdown of FOXO3 at transcription and translation levels in DB and SU-DHL-4 cells. (C) and (D) The expression of RASIP1 was measured after silencing of FOXO3.
