# Supplementary material for: Ras interacting protein 1 facilitated proliferation and invasion of diffuse large B-cell lymphoma cells
Source: Cancer Biol Ther. 2023 Mar 26;24(1):2193114. doi: 10.1080/15384047.2023.2193114 (PMC10054171; doi:10.1080/15384047.2023.2193114)
Supplement: Supplemental Material [file KCBT_A_2193114_SM5303.zip › table S1.docx]

**Table S1. The data presented in this study.**

Figure 1B

|  | DB | SU-DHL-4 |
| --- | --- | --- |
| parental | 1.00±0.00 | 1.00±0.00 |
| pcDNA3.1 | 1.07±0.19 | 0.92±0.10 |
| RASIP1 | 7.29±1.08 | 7.74±0.78 |

Figure 1C-1D

|  | DB | SU-DHL-4 |
| --- | --- | --- |
| parental | 1.00±0.00 | 1.00±0.00 |
| siNC | 1.12±0.12 | 0.94±0.05 |
| siRASIP1-a | 0.29±0.03 | 0.32±0.05 |
| siRASIP1-b | 0.34±0.06 | 0.36±0.04 |
| siRASIP1-c | 0.51±0.09 | 0.48±0.08 |
| siRASIP1-d | 0.41±0.07 | 0.45±0.07 |

Figure 2A

DB

|  | 0 h | 24 h | 48 h | 72 h |
| --- | --- | --- | --- | --- |
| parental | 0.340±0.048 | 0.576±0.069 | 0.792±0.090 | 0.975±0.138 |
| pcDNA3.1 | 0.328±0.048 | 0.602±0.079 | 0.853±0.119 | 1.00±0.149 |
| RASIP1 | 0.346±0.045 | 0.903±0.096 | 1.289±0.136 | 1.625±0.175 |

SU-DHL-4

|  | 0 h | 24 h | 48 h | 72 h |
| --- | --- | --- | --- | --- |
| parental | 0.331±0.037 | 0.542±0.063 | 0.864±0.097 | 0.897±0.055 |
| pcDNA3.1 | 0.329±0.049 | 0.587±0.068 | 0.779±0.038 | 0.855±0.069 |
| RASIP1 | 0.358±0.055 | 0.794±0.098 | 1.355±0.129 | 1.582±0.113 |

Figure 2B

DB

|  | 0 h | 24 h | 48 h | 72 h |
| --- | --- | --- | --- | --- |
| parental | 0.333±0.043 | 0.586±0.079 | 0.791±0.087 | 0.964±0.138 |
| siNC | 0.335±0.038 | 0.591±0.062 | 0.837±0.096 | 1.005±0.119 |
| siRASIP1-1 | 0.325±0.045 | 0.364±0.044 | 0.459±0.052 | 0.550±0.066 |
| siRASIP1-2 | 0.319±0.042 | 0.375±0.038 | 0.497±0.053 | 0.581±0.068 |

SU-DHL-4

|  | 0 h | 24 h | 48 h | 72 h |
| --- | --- | --- | --- | --- |
| parental | 0.334±0.044 | 0.579±0.072 | 0.829±0.076 | 0.886±0.077 |
| siNC | 0.316±0.032 | 0.552±0.064 | 0.870±0.076 | 0.941±0.053 |
| siRASIP1-1 | 0.318±0.044 | 0.335±0.043 | 0.413±0.052 | 0.506±0.069 |
| siRASIP1-2 | 0.308±0.039 | 0.350±0.050 | 0.452±0.060 | 0.503±0.042 |

Figure 2D

DB

|  | G1 | S | G2 |
| --- | --- | --- | --- |
| parental | 61.06±0.754 | 20.84±0.578 | 18.02±0.679 |
| pcDNA3.1 | 61.64±1.924 | 19.90±2.999 | 18.36±2.850 |
| RASIP1 | 47.37±1.126 | 33.47±2.003 | 19.07±0.906 |

SU-DHL-4

|  | G1 | S | G2 |
| --- | --- | --- | --- |
| parental | 57.70±0.512 | 20.94±1.518 | 20.05±1.435 |
| pcDNA3.1 | 58.48±0.923 | 20.32±2.006 | 20.45±1.194 |
| RASIP1 | 41.51±2.142 | 36.59±1.014 | 20.70±0.480 |

Figure 2E

DB

|  | G1 | S | G2 |
| --- | --- | --- | --- |
| parental | 60.25±3.285 | 20.66±3.067 | 18.87±0.420 |
| siNC | 60.95±1.228 | 20.40±1.375 | 18.31±2.357 |
| siRASIP1-1 | 79.06±0.826 | 10.91±0.895 | 9.09±0.403 |
| siRASIP1-2 | 78.25±0.117 | 11.13±0.748 | 9.89±0.774 |

SU-DHL-4

|  | G1 | S | G2 |
| --- | --- | --- | --- |
| parental | 57.75±0.679 | 20.61±1.267 | 20.47±0.881 |
| siNC | 58.15±0.881 | 20.35±0.525 | 20.65±1.647 |
| siRASIP1-1 | 78.52±0.435 | 9.91±0.614 | 10.44±0.778 |
| siRASIP1-2 | 77.12±0.254 | 10.57±0.571 | 11.26±0.601 |

Figure 3B

DB

|  | early | late |
| --- | --- | --- |
| parental | 0.23±0.22% | 1.34±0.23% |
| siNC | 1.38±0.12% | 1.23±0.11% |
| siRASIP1-1 | 23.72±4.71% | 19.39±3.37% |
| siRASIP1-2 | 21.22±1.06% | 19.78±3.37% |

SU-DHL-4

|  | early | late |
| --- | --- | --- |
| parental | 1.56±0.47% | 1.61±0.11% |
| siNC | 1.29±0.57% | 1.42±0.46% |
| siRASIP1-1 | 11.29±1.89% | 31.56±3.87% |
| siRASIP1-2 | 13.45±3.26% | 28.91±2.30% |

Figure 4A

|  | DB | SU-DHL-4 |
| --- | --- | --- |
| parental | 16266.67±2402.78 | 15600.00±1600.00 |
| pcDNA3.1 | 15033.33±1929.59 | 14500.00±1322.88 |
| RASIP1 | 27533.33±4148.90 | 27400.00±4703.19 |

Figure 4B

|  | DB | SU-DHL-4 |
| --- | --- | --- |
| parental | 17833.33±2554.08 | 15733.33±1616.58 |
| siNC | 17533.33±4500.37 | 15933.33±2386.07 |
| siRASIP1-1 | 5800.00±916.52 | 5066.67±832.67 |
| siRASIP1-2 | 6133.33±1803.70 | 5666.67±986.58 |

Figure 5B

|  | DB | SU-DHL-4 |
| --- | --- | --- |
| parental | 1.00±0.00 | 1.00±0.00 |
| pcDNA3.1 | 1.10±0.15 | 0.98±0.10 |
| FOXO3 | 8.29±1.28 | 7.56±0.82 |

Figure 5D

|  | DB | SU-DHL-4 |
| --- | --- | --- |
| parental | 1.00±0.00 | 1.00±0.00 |
| pcDNA3.1 | 0.96±0.13 | 0.96±0.18 |
| FOXO3 | 0.37±0.06 | 0.42±0.07 |

Figure 5F

|  | Seq 1 | Seq 2 | Seq 3 | Seq 4 |
| --- | --- | --- | --- | --- |
| pcDNA3.1 | 0.0584±0.0061 | 0.0654±0.0062 | 0.0589±0.0062 | 0.0549±0.0059 |
| FOXO3 | 0.0197±0.00072 | 0.0234±0.0023 | 0.0267±0.0032 | 0.0501±0.0013 |

Figure 6A

|  | mean±SD |
| --- | --- |
| pcDNA3.1 | 1.00±0.00 |
| FOXO3 | 0.40±0.04 |
| FOXO3+RASIP1 | 2.53±0.41 |

Figure 6B

|  | 0 h | 24 h | 48 h | 72 h |
| --- | --- | --- | --- | --- |
| pcDNA3.1 | 0.332±0.043 | 0.581±0.066 | 0.794±0.085 | 0.982±0.109 |
| FOXO3 | 0.326±0.044 | 0.359±0.044 | 0.465±0.054 | 0.585±0.070 |
| FOXO3+RASIP1 | 0.337±0.035 | 0.557±0.057 | 0.757±0.086 | 0.932±0.103 |

Figure 6D

|  | early | late |
| --- | --- | --- |
| pcDNA3.1 | 1.33±0.11% | 0.84±0.29% |
| FOXO3 | 18.99±3.44% | 22.26±1.41% |
| FOXO3+RASIP1 | 2.42±0.24% | 1.42±0.07% |

Figure 6E

|  | mean±SD |
| --- | --- |
| pcDNA3.1 | 16666.67±3055.05 |
| FOXO3 | 5946.67±903.62 |
| FOXO3+RASIP1 | 14533.33±2203.03 |

Figure 7A

|  | DB | SU-DHL-4 |
| --- | --- | --- |
| shNC | 1.00±0.00 | 1.00±0.00 |
| shRASIP1-1 | 0.25±0.05 | 0.22±0.04 |
| shRASIP1-2 | 0.28±0.05 | 0.26±0.05 |

Figure 7D

DB

|  | 7 | 10 | 13 | 16 | 19 | 22 | 25 | 28 |
| --- | --- | --- | --- | --- | --- | --- | --- | --- |
| shNC | 10.85±8.67 | 22.33±9.14 | 53.19±21.19 | 120.32±29.52 | 213.78±36.56 | 332.49±46.30 | 484.78±74.61 | 642.91±98.24 |
| shRASIP1-1 | 11.54±4.47 | 21.87±5.86 | 30.37±6.01 | 44.25±3.97 | 66.55±10.96 | 86.00±13.24 | 101.05±19.99 | 125.02±25.03 |
| shRASIP1-2 | 13.78±5.84 | 20.40±9.68 | 33.81±9.97 | 45.65±15.03 | 62.72±23.33 | 79.74±30.86 | 95.20±40.20 | 115.22±19.95 |

SU-DHL-4

|  | 7 | 10 | 13 | 16 | 19 | 22 | 25 | 28 |
| --- | --- | --- | --- | --- | --- | --- | --- | --- |
| shNC | 11.09±1.94 | 48.38±23.01 | 91.19±29.39 | 170.64±61.17 | 246.33±93.50 | 375.19±143.72 | 477.67±162.41 | 609.98±213.90 |
| shRASIP1-1 | 11.02±3.13 | 15.83±3.71 | 31.60±6.72 | 51.71±11.37 | 75.18±11.00 | 96.81±24.33 | 112.75±31.89 | 134.18±32.57 |
| shRASIP1-2 | 11.38±2.91 | 16.15±4.13 | 24.99±7.37 | 40.30±13.32 | 53.67±19.03 | 66.71±18.12 | 78.51±22.22 | 96.38±24.33 |

Figure 7E

|  | DB | SU-DHL-4 |
| --- | --- | --- |
| shNC | 618.50±101.48 | 592.83±215.89 |
| shRASIP1-1 | 118.33±23.44 | 123.50±29.06 |
| shRASIP1-2 | 111.50±48.12 | 90.83±21.95 |

Figure S1A

|  | mean±SD |
| --- | --- |
| primary B cells | 1.00±0.00 |
| DB | 3.07±0.44 |
| SU-DHL-4 | 2.13±0.36 |

Figure S2A

|  | DB | SU-DHL-4 |
| --- | --- | --- |
| siNC | 1.00±0.00 | 1.00±0.00 |
| siFOXO3 | 0.31±0.05 | 0.27±0.03 |

Figure S2C

|  | DB | SU-DHL-4 |
| --- | --- | --- |
| siNC | 1.00±0.00 | 1.00±0.00 |
| siFOXO3 | 1.88±0.30 | 2.02±0.32 |
