# Supplementary figures and images for: Ras interacting protein 1 facilitated proliferation and invasion of diffuse large B-cell lymphoma cells
Source: Cancer Biol Ther. 2023 Mar 26;24(1):2193114. doi: 10.1080/15384047.2023.2193114 (PMC10054171; doi:10.1080/15384047.2023.2193114)

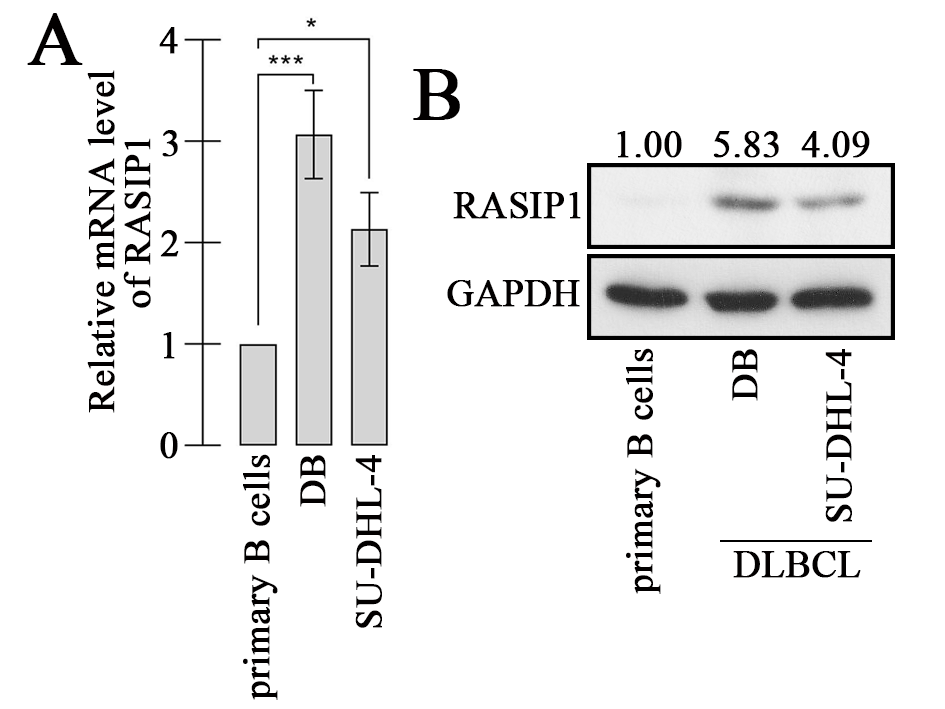

Supplement: Supplemental Material [file KCBT_A_2193114_SM5303.zip › figure S1 (1).tif]

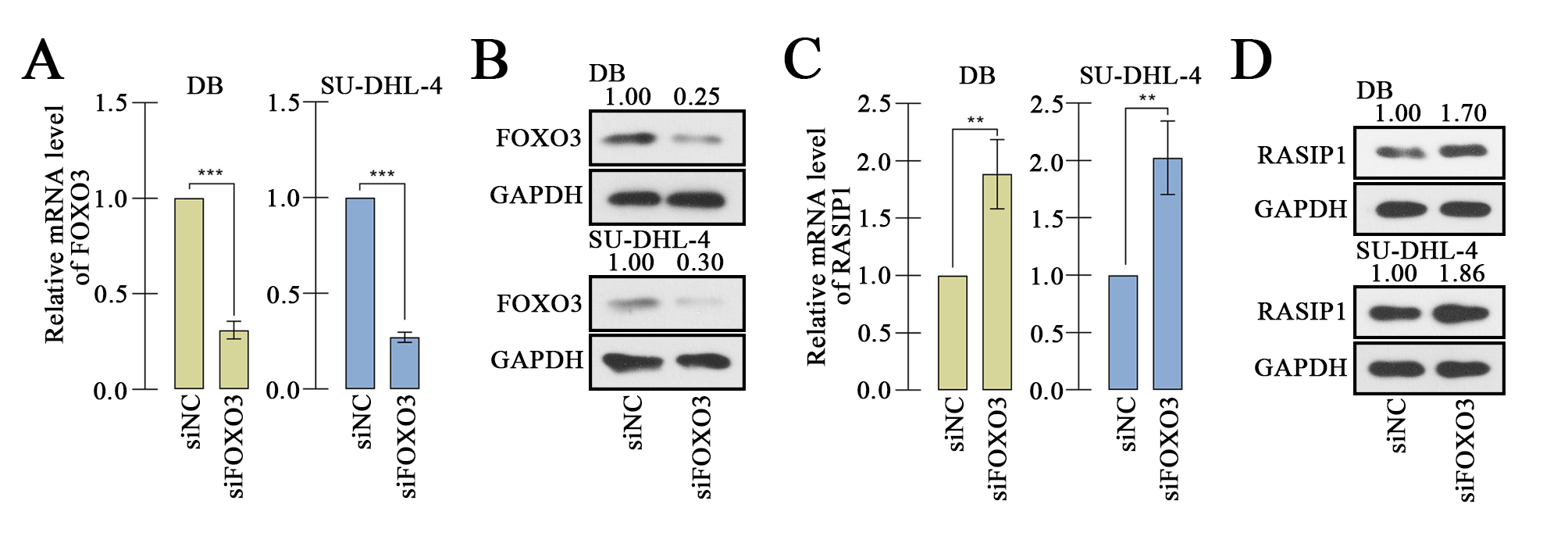

Supplement: Supplemental Material [file KCBT_A_2193114_SM5303.zip › figure S2 (1).tif]
